# Whole-Genome Draft Sequences of Nine Asymptomatic *Escherichia coli* Bacteriuria Isolates from Diabetic Patients

**DOI:** 10.1128/genomeA.01369-17

**Published:** 2018-01-11

**Authors:** Christoph Stork, Beáta Kovács, Eva Trost, Tamás Kovács, György Schneider, Barnabás Rózsai, Monika Kerényi, Levente Emődy, Ulrich Dobrindt

**Affiliations:** a Institute of Hygiene, University of Münster, Münster, Germany; b Department of Medical Microbiology and Immunology, University of Pécs, Pécs, Hungary; c 1st Department of Medicine, University of Pécs, Pécs, Hungary; d Nanophagetherapy Center, Enviroinvest Corp., Pécs, Hungary; e Department of Pediatrics, University of Pécs, Pécs, Hungary

## Abstract

*Escherichia coli* can colonize the urinary bladder without causing a disease response in the host. This asymptomatic bacteriuria (ABU) can protect against recurrent symptomatic urinary tract infection by virulent bacteria. Here, we report the whole-genome sequences of nine *E. coli* ABU isolates from diabetic patients.

## GENOME ANNOUNCEMENT

Urinary tract infection (UTI) constitutes a serious global health problem associated with considerable morbidity and death and can affect patient quality of life. Symptomatic UTI is caused most often by *Escherichia coli*. Besides provoking symptoms, *E. coli* can also asymptomatically colonize the human bladder for extended time periods. Asymptomatic bacteriuria (ABU) does not, with some exceptions, require any treatment (1). Deliberate colonization of the urinary bladder thus represents a promising alternative to antibiotic treatment and prevents colonization by more virulent bacterial strains (2–4). To characterize bacterial traits that contribute to the fitness and competitiveness of *E. coli* ABU strains in urine, we sequenced nine ABU isolates from patients with diabetes mellitus in Hungary.

Total genomic DNA was isolated from the *E. coli* ABU isolates using the MagAttract high-molecular-weight (HMW) DNA kit (Qiagen, Hilden, Germany). To prepare 500-bp paired-end libraries of all ABU isolates, we used the Nextera XT DNA library preparation kit (Illumina, San Diego, CA, USA). Libraries were sequenced on the Illumina MiSeq sequencing platform using v2 sequencing chemistry. *E. coli* ABU isolate 65 was sequenced twice on the Illumina platform. In addition, this strain was subjected to whole-genome bidirectional sequencing using the Roche GS Junior sequencer with titanium chemistry (Roche Hungary Ltd., Budaörs, Hungary). *E. coli* ABU strain 65 reads from the Illumina and Roche sequencing platforms were included in a hybrid assembly. Prior to assembly, raw reads of all ABU isolates were quality checked with FastQC (v11.5) (http://www.bioinformatics.babraham.ac.uk/projects/fastqc), and low-quality reads were trimmed using Sickle (v1.33) (https://github.com/najoshi/sickle). Subsequently, the quality-filtered reads were *de novo* assembled using SPAdes (v3.9.0) (5). Final genome assemblies were analyzed using QUAST (v4.3) (6), resulting in 43 to 197 contigs of >1 kb, and accounting for total genome sizes ranging from 4,578,015 to 5,285,335 bp (Table 1). Coding sequences (CDSs) and orthologous genes were determined using Prokka (v1.12) (7) and Proteinortho (v5.15) (8). Between 4,204 and 5,035 coding DNA sequences were identified in the corresponding genomes (Table 1). To predict and identify plasmids, serotypes, and acquired antibiotic resistance mechanisms, we performed a systematic analysis, querying the databases PlasmidFinder (v1.3) (9), SerotypeFinder (v1.1) (10), and ResFinder (v2.1) (11), respectively. For the determination of virulence factors (VFs), we extended the freely available *E. coli* VF collection (v0.1) (https://doi.org/10.5281/zenodo.56686). Core genome multilocus sequence typing (cgMLST) was performed with SeqSphere^+^ (v3.4.0) (Ridom GmbH, Münster, Germany).

**TABLE 1:** Features and assembly metrics of the genome sequences of nine *E. coli* ABU isolates from diabetic patients

*E. coli* strain	ECOR phylogroup (ST)^a^	No. of raw paired reads	No. of trimmed reads	No. of contigs	Genome size (bp)	*N*_50_ (bp)	*L*_50_	Coverage (×)	No. of CDSs^b^	GenBank accession no.
ABU 1	B1 (205)	11,310,408	8,774,314	43	4,578,015	236,117	8	285	4,204	PENN00000000
ABU 9	B2 (95)	10,142,410	7,982,006	106	5,212,357	220,362	7	228	4,862	PENO00000000
ABU 61	F (59)	6,927,866	3,800,294	197	5,183,452	55,491	28	158	4,888	PENP00000000
ABU 65	F (117)	5,847,714,^c^ 230,941^d^	4,315,886,^c^ 217,712^d^	124	5,184,119	150,223	12	179,^c^ 16^d^	5,035	PENQ00000000
ABU 84	B2 (73)	9,577,806	7,435,548	78	5,223,185	301,227	6	212	4,871	PENR00000000
ABU 91	B2 (95)	8,845,416	6,756,004	81	5,285,335	268,769	7	190	5,002	PENJ00000000
ABU 106	B2 (95)	1,220,066	774,552	108	4,939,518	129,842	10	33	4,601	PENK00000000
ABU 123	D (69)	6,759,080	2,783,684	96	4,917,555	115,220	13	116	4,547	PENL00000000
ABU 148	B2 (73)	9,488,588	7,435,092	87	5,154,177	248,638	7	214	4,795	PENM00000000

a ECOR, *E. coli* Reference Collection; ST, sequence type.

b CDS, coding sequence.

c Combined Illumina reads.

d 454 reads.

The draft genomes of nine clinical ABU isolates from diabetic patients will serve as a useful source for upcoming comparative studies of *E. coli* isolate virulence- and fitness-related traits that promote efficient growth and competitiveness in urine. The identification of bacterial characteristics relevant for efficient and safe therapeutic bladder colonization will improve the application of bacterial interference.

### Accession number(s).

The assembled draft genome sequences obtained from this project were deposited into DDBJ/ENA/GenBank under the accession numbers listed in Table 1.
